# Synchronous detection method for senescence quality of damaged Korla fragrant pears during storage

**DOI:** 10.3389/fpls.2025.1726333

**Published:** 2026-02-05

**Authors:** Jingchi Guo, Hao Niu, Yang Liu, Quan Xu, Haonan Xue, Haipeng Lan, Shengkun Dong, Yifei Gao

**Affiliations:** 1 Modern Agricultural Engineering Key Laboratory at Universities of Education Department of Xinjiang Uygur Autonomous Region, Tarim University, Alaer, China; 2 Xinjiang Production and Construction Corps Key Laboratory of Utilization and Equipment of Special, Agricultural and Forestry Products in Southern Xinjiang, Alaer, China; 3 College of Mechanical and Electronic Engineering, Tarim University, Alaer, China

**Keywords:** Korla fragrant pears, senescence, synchronous detection, damage, storage

## Abstract

**Introduction:**

Korla fragrant pears are highly susceptible to mechanical damage during harvest, storage, and transport, which accelerates fruit browning and senescence, leading to fruit degradation and even a complete loss of commercial value.

**Methods:**

To enhance the utilization value of damaged pears, this study used superoxide dismutase (SOD) activity, catalase (CAT) activity, peroxidase (POD) activity, superoxide anion ( $O_{2}^{-.}$) generation rate, and hydrogen peroxide (H_2_O_2_) content—factors directly related to pear browning—as evaluation indicators of senescence quality, and investigated the changes in the senescence quality of damaged pears with varying injury levels under impact load during storage. Furthermore, a multi-output model for predicting the senescence quality of damaged pears during storage was constructed using partial least squares regression (PLSR), support vector regression (SVR), and long short-term memory (LSTM). The optimal prediction model was subsequently selected from these.

**Results:**

The results indicated that as storage time increased, the average SOD activity, CAT activity, POD activity, O2^-.^ generation rate, and H_2_O_2_ content in pears with different injury levels gradually increased. Higher damage levels resulted in a more rapid change rate of senescence quality. The constructed SVR multi-output model was the optimal model for predicting the senescence quality of damaged pears during storage, achieving R^2^ values above 0.95 for the prediction of SOD activity, CAT activity, POD activity, O2^-.^ generation rate, and H_2_O_2_ content.

**Discussion:**

These findings provide a theoretical reference for investigating fruit senescence mechanisms and the synchronous detection of senescence quality.

## 1 Introduction

Global population growth is accompanied by a year-on-year increase in consumer demand for fresh fruits and vegetables. Fruit quality is crucial for consumer health and daily dietary expectations (Adaskaveg and Blanco-Ulate, 2023; Ali et al., 2021; Benashvili et al., 2025). The Korla fragrant pear (*Pyrus sinkiangensis Yü*) is a characteristic ancient pear variety from Xinjiang, China, known for its thin skin, fine texture, crispness, juiciness, sweet flavor, and high nutritional value, giving it strong market appeal (An et al., 2023; Liu et al., 2021; Wang et al., 2021). However, these pears are susceptible to varying degrees of mechanical damage during harvesting, transportation, and post-harvest handling. The internal tissues at the injury sites are prone to browning, which directly affects the fruit’s visual appeal, shelf life, and market value (Goldental-Cohen et al., 2019; Yuan et al., 2025; Zhu et al., 2024). Industry practitioners often discard damaged fruit before storage, leading to direct economic losses of 25%-45% and significantly hindering the development of the Korla fragrant pear industry (Liu et al., 2023). As a major abiotic stress, mechanical damage stimulates the production of reactive oxygen species (ROS) at the wound site. ROS metabolism can lead to the formation of lignin and suberin, promoting wound healing. This self-healing capacity and protective mechanism mean that some damaged pears retain commercial value (Yu et al., 2021). Nevertheless, excessive ROS accumulation can destroy cell membrane structures, accelerate fruit browning and senescence, and impair fruit quality. Selling the damaged fruit before the onset of browning and senescence can increase growers’ income and avoid resource waste (Dong et al., 2021). Therefore, elucidating the changes in senescence indicators, such as ROS, in damaged Korla fragrant pears during storage, and constructing an efficient and reliable prediction model for senescence quality, can provide theoretical guidance for controlling storage quality and determining the optimal time for removal from storage. This has significant practical implications for advancing the Korla fragrant pear industry.

ROS are a collective term for oxygen-containing, chemically reactive molecules in living organisms, primarily including $O_{2}^{-.}$, H_2_O_2_, among others (Wei et al., 2025). The metabolism and accumulation of $O_{2}^{-.}$ and H_2_O_2_ are directly associated with fruit browning and are generated by various cellular processes, such as photosynthesis and respiration, occurring in multiple organelles (Adiletta et al., 2021). Under mechanical damage stress, fruits undergo oxidative stress. Elevated ROS levels induce membrane lipid peroxidation, protein denaturation, disruption of cellular structure and function, thereby accelerating fruit senescence and quality deterioration (Mittler et al., 2022). Antioxidant enzymes, including SOD, CAT, and POD, play a crucial role in inducing and scavenging ROS. Maintaining a balance between ROS production and elimination can delay fruit senescence (Gan et al., 2024). Therefore, SOD activity, CAT activity, POD activity, $O_{2}^{-.}$ generation, and H_2_O_2_ content serve as key physiological indicators reflecting the fruit senescence process. By maintaining the internal ROS metabolic balance, they play a crucial role in regulating fruit growth, development, and aging. The patterns of quality changes in fruit senescence under damage stress have garnered significant attention from researchers. Guan et al. (2025). investigated the changes in ROS and degrading enzyme activities in mushrooms during storage under vibration conditions. They showed that $O_{2}^{-.}$ and H_2_O_2_ levels initially increased and then gradually decreased, while SOD and CAT activities overall increased post-vibration before declining in the subsequent days. Ackah et al. (2022) demonstrated that mechanical damage in fruits triggers ROS formation, which subsequently weakens cell walls and causes fruit damage. Castro-Mercado et al. (2009) found that cutting injury induced local oxidative bursts in immature ‘Hass’ avocados, with superoxide anion levels rising within 15 minutes, lipid peroxidation occurring after 2 hours, and H_2_O_2_ concentration and peroxidase activity decreasing with distance after 24 hours. Li et al. (2017) studied the response of phospholipase D and the antioxidant system to mechanical injury in postharvest banana fruits. They reported that the activities of antioxidant enzymes such as SOD, CAT, and POD were significantly enhanced in damaged bananas during storage, and mechanical injury increased the content of ROS, including $O_{2}^{-.}$ and H_2_O_2_. These studies collectively indicate that mechanical damage significantly influences the senescence quality of fruits during storage. Furthermore, detecting and controlling the senescence quality of fragrant pears during storage aids in optimizing storage techniques and reducing postharvest loss rates. Traditional methods for assessing fruit senescence quality are often inefficient and inaccurate, highlighting the need for scientific and efficient approaches to evaluate the internal quality of Korla fragrant pears during storage.

The rapid development of machine learning and deep learning has enabled efficient detection of fruit quality (Ahmed et al., 2025). Razavi et al. (2025) investigated the changes in POD activity associated with browning in ‘Red Delicious’ apples during storage. The forest optimization algorithm combined with an artificial neural network achieved an R^2^ of 0.99 for predicting POD and an R^2^ of 6.69 for predicting RPD in the testing phase. Zhang et al. (2022) developed a quantitative prediction model for CAT in wheat, demonstrating that a support vector machine model trained on data preprocessed with multiplicative scatter correction and the successive projections algorithm yielded the best prediction performance, with an R^2^ of 0.9664. Lopes et al. (2020) efficiently detected changes in the secondary and tertiary structures of horseradish POD using partial least squares regression. The non-destructive methods developed in these studies for assessing the senescence quality of agricultural products have achieved satisfactory prediction performance. However, current research on non-destructive detection of product quality predominantly focuses on the development and application of models for single indicators. The quality evaluation of agricultural products often requires the integration of multiple key indicators. Single-indicator detection methods are inefficient for multi-indicator prediction tasks, involve high model construction and maintenance costs, and cannot provide a comprehensive and accurate assessment of the overall quality of fruits (Guo et al., 2025). To address the limitations of single-indicator assessments in comprehensive quality evaluation, synchronous detection methods for multiple indicators have been extensively explored by researchers. Peng et al. (2025) accurately predicted the protein, fat, moisture content, and acidity of goat milk powder using a random forest-based synchronous detection method, with correlation coefficients R of 0.9846, 0.9642, 0.9915, and 0.9819, respectively. Ouyang et al. (2021) achieved synchronous detection of multiple chemical components in matcha using a multi-output structure based on the partial least squares algorithm, reporting R values of 0.8077, 0.7098, 0.7942, and 0.8473 for predicting caffeine, tea polyphenols, free amino acids, and chlorophyll, respectively. The synchronous detection methods constructed in the aforementioned studies enable the simultaneous prediction of multiple quality indicators of agricultural products, significantly improving detection efficiency. Nevertheless, research on synchronous detection methods for the senescence quality of Korla fragrant pears during storage under mechanical damage stress has not been reported.

This study regarded the SOD activity, CAT activity, POD activity, $O_{2}^{-.}$ generation rate, and H_2_O_2_ content, which are directly related to pear browning, as evaluation indicators of senescence quality. It investigated the change patterns of senescence quality in Korla fragrant pears with different damage levels during storage. Based on PLSR, SVR, and LSTM multi-output models, a synchronous detection method was established for measuring SOD activity, CAT activity, POD activity, $O_{2}^{-.}$ generation rate, and H_2_O_2_ content in damaged fragrant pears during storage. The optimal prediction model was selected, providing theoretical guidance for the efficient multi-index detection of fruits.

## 2 Materials and methods

### 2.1 Sample collection

All Korla fragrant pear samples used in this study were collected from an orchard under conventional management at Company 10, Regiment 10, Alaer City, affiliated with the First Division of the Xinjiang Production and Construction Corps. The pears were harvested on September 15, 2023. Fruits that were free from deformities or scars, pests or diseases, with smooth surfaces and uniform size (130 ± 5 g), were selected for the experiments. All personnel involved in harvesting wore gloves and carefully detached the fruits at the stalk. After harvesting, the fruits were placed in foam nets and packaged in boxes to prevent mechanical damage during collection and were transported to the laboratory on the same day.

### 2.2 Impact damage experiment on Korla fragrant pears

**Figure 1:**
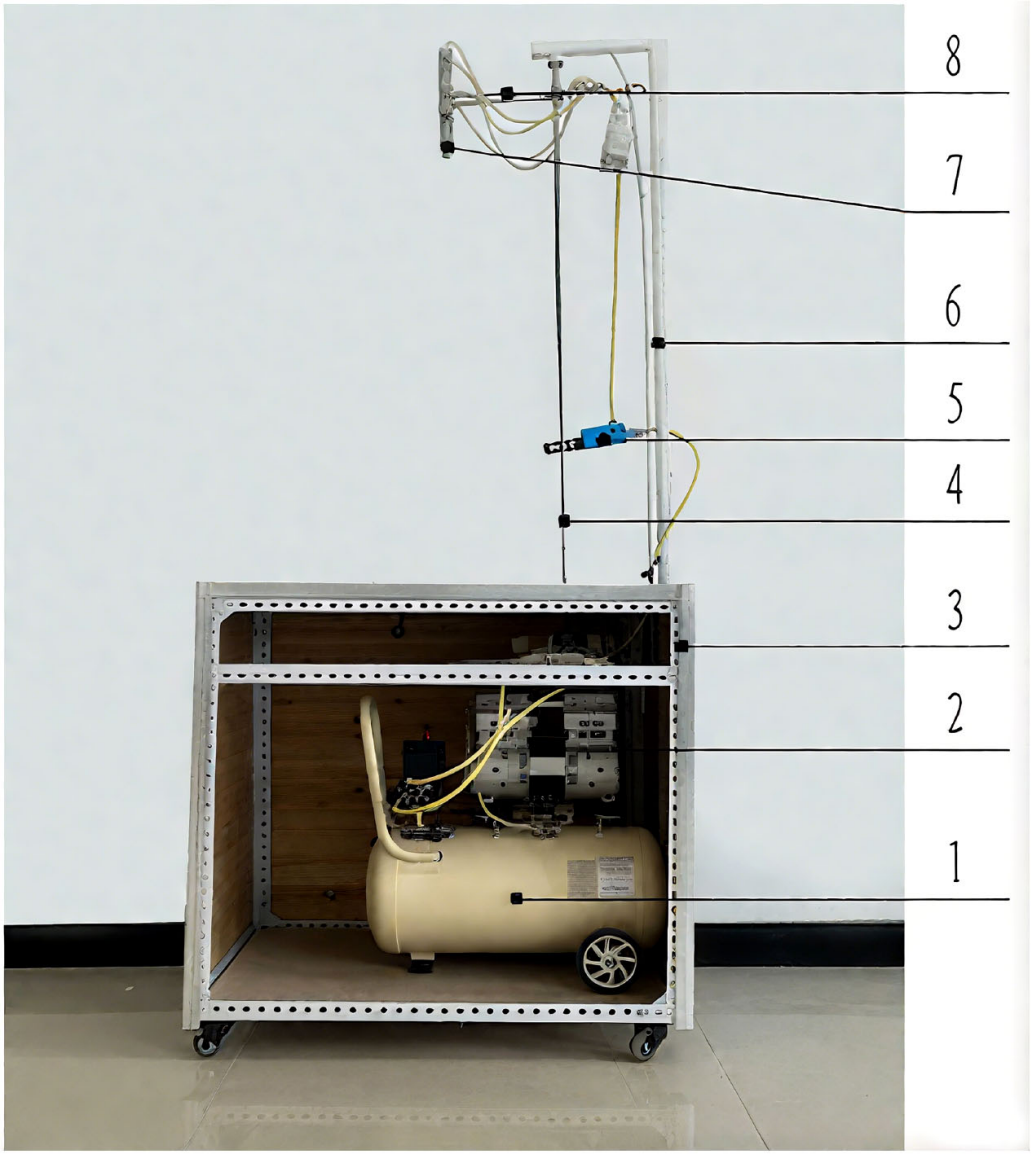
Impact damage test bench for fragrant pears (**1.** Air compressor **2.** Engine body **3.** Frame **4.** Screw **5.** Vacuum generator **6.** Linear guide **7.** Suction cup **8.** Cantilever).

In this study, impact damage tests on Korla fragrant pears were performed using a custom-built test bench (**Figure 1**). The test bench comprised a lifting device and an adsorption device. The lifting device included a screw, a linear guide, a pneumatic motor, and a cantilever, while the adsorption device consisted of a vacuum generator, a suction cup, and an air compressor. The vacuum suction cup on the cantilever was raised to a predetermined height along the calibrated linear guide via the lifting device. The adsorption device was then used to secure the pear sample onto the suction cup. Subsequently, the adsorption device was deactivated, allowing the sample to fall freely from the suction cup, thus completing the impact damage test.

Prior to the formal experiment on Korla fragrant pears, preliminary tests were conducted. At an impact height of 276 mm, the pears sustained slight latent damage that was barely visible to the naked eye, with an average measured damage volume of 905 mm^3^. When the impact height was increased to 340 mm, the pears exhibited moderate latent damage, characterized by minor surface marks and browning in the flesh after peeling, corresponding to an average damage volume of 1665 mm^3^. At an impact height of 398 mm, the pear samples developed shallow surface scars under the mechanical load, and the internal flesh began to deteriorate, indicating slight apparent damage, with an average damage volume of 2411 mm^3^. When the impact height reached 535 mm, the pear samples showed obvious surface damage, and the flesh was significantly damaged upon peeling, although the skin remained unbroken, resulting in an average damage volume of 3765 mm^3^. At an impact height of 670 mm, the skin of the pears ruptured with juice exudation, and the flesh was extensively damaged, yielding an average damage volume of 5126 mm^3^. In these preliminary tests, each impact height was tested with 10 replicates to obtain the average damage volume.

Therefore, in this experiment, pears subjected to impact load damage were categorized into six damage levels based on damage volumes of 0 mm^3^ (i.e., undamaged pears), 905 mm^3^, 1665 mm^3^, 2411 mm^3^, 3765 mm^3^, and 5126 mm^3^, to investigate the effects of different damage levels on the aging quality of fragrant pears. The impact height was set at 0 mm, 276 mm, 340 mm, 398 mm, 535 mm, and 670 mm. For each damage volume level, 100 damaged pear samples were collected for the storage experiment.

### 2.3 Detection of senescence-related factors in fruits

#### 2.3.1 Detection of superoxide dismutase activity

Superoxide dismutase (SOD, EC 1.15.1.1) catalyzes the dismutation of the superoxide anion into hydrogen peroxide and water, thereby scavenging superoxide anion radicals and protecting cells from oxidative damage (Wang et al., 2018). SOD activity was determined according to the method described by Caracciolo et al. (2020). Approximately 0.1 g of tissue was weighed and homogenized in 1 mL of extraction buffer at 4°C. The homogenate was centrifuged at 12,000 rpm for 10 min at 4°C, and the supernatant was collected as the test solution. The reaction mixture consisted of 50 mM potassium phosphate buffer, 0.1 mM EDTA-Na, 13 mM methionine, 75 μM nitrobluetetrazolium chloride, 75 μM riboflavin, and the crude enzyme extract. After thorough mixing, the solution was incubated at room temperature (25 °C) in the dark for 30 min, and the absorbance of each well was immediately measured at 450 nm using a microplate reader. The inhibition rate of SOD on the WST-8 reduction reaction by superoxide anions was calculated, and the SOD activity in the sample was determined based on the SOD standard curve. One unit of SOD activity (U/g) was defined as the amount of enzyme that caused 50% inhibition of the superoxide anion-mediated reduction reaction in the xanthine oxidase-coupled system described above.

#### 2.3.2 Detection of catalase activity

Catalase (CAT, EC 1.11.1.6) is involved in ROS metabolism and defense responses. Measuring the CAT activity in fruits provides insights into their physiological status, maturity, and postharvest quality changes (Li et al., 2020). The CAT activity was determined following the method described by Chai et al. (2021) with slight modifications. Approximately 0.1 g of tissue was weighed and homogenized with 1 mL of extraction buffer in an ice bath. The homogenate was centrifuged at 12,000 rpm for 10 min at 4 °C and the supernatant was collected and kept on ice for subsequent analysis. A mixture was prepared by sequentially adding potassium dihydrogen phosphate buffer (100 mM; pH 7.0), hydrogen peroxide (20 mM), and the crude enzyme extract. After thorough mixing, 10 μL of the mixture was immediately transferred to a spectrophotometer to record the absorbance at 510 nm, and the CAT enzyme activity was calculated. One unit (U) of enzyme activity was defined as the amount of enzyme required to catalyze the decomposition of 1 μmol of H_2_O_2_ per minute per mL of reaction mixture at 25°C.

#### 2.3.3 Detection of peroxidase activity

Peroxidase (POD, EC 1.11.1.7) is a crucial oxidoreductase, whose activity level is closely associated with fruit resistance (Xu et al., 2024). The POD activity was determined according to the method described by Modesti et al. (2018) Briefly, approximately 0.1 g of tissue was weighed, and 1 mL of extraction buffer was added for homogenization in an ice bath. The homogenate was centrifuged at 12,000 rpm for 10 min at 4°C. The resulting supernatant was collected and kept on ice for subsequent assay. The reaction mixture in the microplate well consisted of potassium phosphate buffer (0.1 mM, pH 7.0), EDTA-Na (0.15 mM, pH 7.0), H_2_O_2_ (6.66 mM), guaiacol (8 mM), and the crude enzyme extract. In the presence of H_2_O_2_, POD catalyzes the oxidation of guaiacol, producing a tan-colored product with a maximum absorbance at 470 nm, which was measured using a spectrophotometer. One unit (U) of enzyme activity was defined as the amount causing an increase of 1 in absorbance at 470 nm per minute per gram of tissue in the reaction system.

#### 2.3.4 Detection of superoxide anion generation rate

Superoxide anion is a type of ROS in organisms and plays important roles in cellular signal transduction, immune responses, and redox balance. However, its excessive production can lead to oxidative stress, thereby damaging cells and tissues. When fruits are subjected to external stress, $O_{2}^{-.}$ are generated and accumulate in large quantities, which can serve as a signal of oxidative stress in the fruit (Liu et al., 2013). The $O_{2}^{-.}$ generation rate was determined according to the method described by Lin et al. (2015) with minor modifications. Approximately 0.1 g of tissue was weighed, and 1 mL of extraction buffer was added. The mixture was homogenized in an ice bath and then centrifuged at 12,000 rpm and 4°C for 10 minutes. The supernatant was collected as the crude enzyme extract and kept on ice for subsequent analysis. Then, 10 mL of phosphate buffer (pH 7.8) was added to the crude enzyme extract and mixed thoroughly. The reaction was carried out at 37°C for 10 minutes. Subsequently, 10 mM guaiacol solution and 10 mM hydrogen peroxide solution were added sequentially and mixed. After reacting at 37°C for 5 minutes, the absorbance was immediately measured at 540 nm using a spectrophotometer. The $O_{2}^{-.}$ generation rate was calculated and expressed as nmol/min/g fresh weight (FW).

#### 2.3.5 Detection of hydrogen peroxide content

Hydrogen peroxide is a key ROS that plays a critical role in plant metabolism. Its content is closely related to fruit ripening, senescence, and stress resistance (Huang et al., 2022). H_2_O_2_ extraction was conducted following the method of Li et al. (2020). Approximately 0.1 g of tissue was weighed, homogenized in 1 mL of acetone in an ice bath, and transferred to a centrifuge tube. The volume was then made up to 1 mL with acetone, and the mixture was centrifuged at 12,000 rpm at 4°C for 10 min. The supernatant was collected and stored on ice for further analysis. A 2 mL aliquot of the supernatant was mixed with 40 μL of titanium tetrachloride and 50 μL of concentrated ammonia. After thorough mixing, the solution was centrifuged at 12,000 rpm and 25°C for 10 min. The resulting yellow precipitate was dissolved in 3.0 mL of sulfuric acid (1.0 mmol·L^-1^). The absorbance of the product was measured at 415 nm using a spectrophotometer to determine the H_2_O_2_ content (μmol·g^-1^ fresh weight).

### 2.4 Korla fragrant pear storage test

To investigate the changes in the senescence-related quality (SOD activity, CAT activity, POD activity, $O_{2}^{-.}$ generation rate, and H_2_O_2_ content) of Korla fragrant pears under different damage levels during shelf life, a storage test on damaged Korla fragrant pears was conducted at the Key Laboratory of Modern Agricultural Engineering, Tarim University, Xinjiang. Pear samples subjected to impact damage were divided into six groups based on different damage levels (damage volumes of 905 mm^3^, 1665 mm^3^, 2411 mm^3^, 3765 mm^3^, 5126 mm^3^, and an undamaged control) and stored at room temperature (Average temperature: 15°C). Each group consisted of 90 samples, resulting in a total of 540 damaged pears collected. The storage period lasted 40 days. Every 5 days, 10 pear samples from each damage level were randomly selected, and measurements of SOD activity, CAT activity, POD activity, $O_{2}^{-.}$ generation rate, and H_2_O_2_ content were performed. The experimental results were averaged.

### 2.5 Construction of a multi-output prediction model for Korla fragrant pear

#### 2.5.1 PLSR

The PLSR multi-output model is a multivariate statistical method based on latent variables, commonly employed in scenarios where both independent variables (X) and dependent variables (Y) are high-dimensional, exhibit multicollinearity, and the sample size is limited (Wan et al., 2022). This method extends the PLSR algorithm by extracting latent co-linear components from both input and output variable sets to establish a regression relationship (Rajendra et al., 2025). Its core principle involves simultaneously decomposing the X and Y matrices to extract a smaller number of latent variables that possess the strongest explanatory power, thereby capturing the covariance information between X and Y to the greatest extent (Wold et al., 2001). A regression model is established between the latent variables of X and those of Y, and this relationship is subsequently mapped back to the original variable space, enabling the simultaneous prediction of multiple outputs Y.

#### 2.5.2 SVR

The SVR multi-output model extends the classical SVR from a single-output to a multi-output form (Cantillo-Luna et al., 2022). Its core idea is to identify an optimal hyperplane in a high-dimensional feature space, thereby minimizing the deviation between the predicted and true values for all output variables. This is achieved by utilizing a kernel function to map the original data into a high-dimensional space, facilitating the identification of an optimal regression model for complex data distributions (Bai et al., 2022). Typically, it accomplishes multi-target prediction by constructing independent models for each output, making it particularly suitable for small-to-medium-scale, multi-output regression problems that require complex, nonlinear mapping.

#### 2.5.3 LSTM

As a variant of recurrent neural networks, LSTM was primarily designed to address the “long-term dependency” problem in traditional recurrent networks, which arises from vanishing or exploding gradients when processing long sequence data. It is specifically used for tasks involving time-series data (Roy et al., 2022). The LSTM multi-output model can capture complex long-term temporal dependencies and dynamic patterns within the data, and directly produce multivariate outputs without requiring complex feature engineering. The core structure of LSTM includes the forget gate $f_{t}$, input gate $i_{t}$, and output gate $o_{t}$, which selectively remember and forget incoming information through these three gating units (Geng et al., 2023). By setting the number of neurons in the fully connected layer to match the number of output variables, multiple related target variables can be predicted directly in a single step. This end-to-end architecture enables automatic feature extraction from raw sequence data and simultaneous prediction.

### 2.6 Calculation of damage volume in fragrant pears

**Figure 2:**
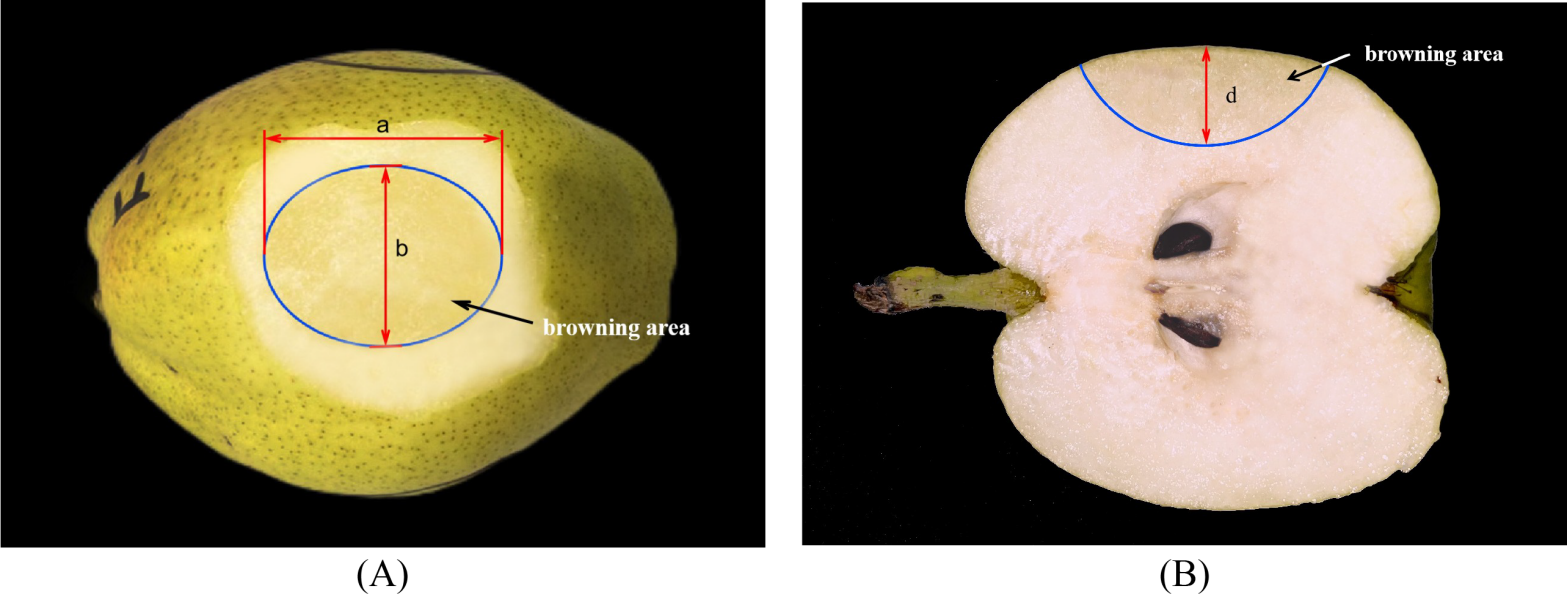
Schematic diagram of mechanical injury on a peeled Korla fragrant pear **(A)** Surface area **(B)** Depth profile.

To visualize the damaged area of the samples clearly, the Korla fragrant pear samples damaged under impact load were placed at room temperature for 24 hours. After peeling, the damaged area exhibited significant browning, with the browning region approximating an ellipse (**Figure 2A**). To better quantify the damage volume, according to the method of Guo et al. (2025), the entire damaged tissue can be considered as part of an ellipsoid. The major axis a and minor axis b of the damaged area were measured using an electronic vernier caliper. The fruit was cut into two equal parts along the stem-calyx axis to obtain a cross-sectional schematic of the damaged area (**Figure 2B**). Subsequently, the depth d of the browning area in the pear cross-section was measured. The damage volume was calculated using Equation 1.

(1) $$\text{V}=\frac{\text{πd}}{24}(3\text{ab}+4{\text{d}}^{2})$$

In the formula, a is the major axis of the fruit damage (mm), b is the minor axis (mm), and d is the depth of the damaged area (mm).

### 2.7 Model evaluation

This study employed R^2^, RMSE, and RPD as criteria for both model parameter selection and model performance evaluation, aiming to identify the optimal prediction model for the quality of damaged fragrant pears. The calculations for R^2^, RMSE, and RPD are provided in Equations 2–4, respectively.

(2) $$R^{2}=1-\frac{\sum_{i=1}^{n}{\left({\hat{y}}_{i}-y_{i}\right)}^{2}}{\sum_{i=1}^{n}{\left(\bar{y}-y_{i}\right)}^{2}}$$

(3) $$RMSE=\sqrt{\frac{1}{n}\sum_{i=1}^{n}{\left(y_{i}-{\hat{y}}_{i}\right)}^{2}}$$

(4) $$RPD=\frac{SD}{RMSE}$$

(5) $$SD=\sqrt{\frac{\sum_{i-1}^{n}{\left(\bar{y}-y_{i}\right)}^{2}}{n-1}}$$

Where SD is the standard deviation of the test set for the analysis samples, *n* is the sample size, ${\hat{y}}_{i}$ denotes the predicted value of the *i*-th sample (see Equation 5 for the calculation method), ${\text{y}}_{\text{i}}$ denotes the actual value of the *i*-th sample, and $\bar{y}$ denotes the mean of the actual values. An R^2^ value closer to 1 indicates a better model fit, conversely, a value further from 1 indicates a poorer fit. A smaller RMSE value indicates a smaller average deviation between the predicted and actual values, denoting higher predictive accuracy of the model. RPD is a widely adopted metric in chemometrics for evaluating model performance. An RPD > 2.5 indicates excellent model validity with high predictive accuracy, 2.0< RPD ≤ 2.5 indicates acceptable model performance for initial screening, and RPD< 1.4 indicates insufficient model reliability, rendering it unsuitable for analytical purposes (Wang et al., 2022).

All linear fitting analyses and plotting involved in this study were performed using Origin (2024) software. Linear regression fitting was conducted between the true values and predicted values, and both two-sided confidence bands and prediction bands were calculated and plotted, with the significance level set at α = 0.05.

## 3 Result and analysis

### 3.1 Changes in superoxide dismutase activity

**Figure 3:**
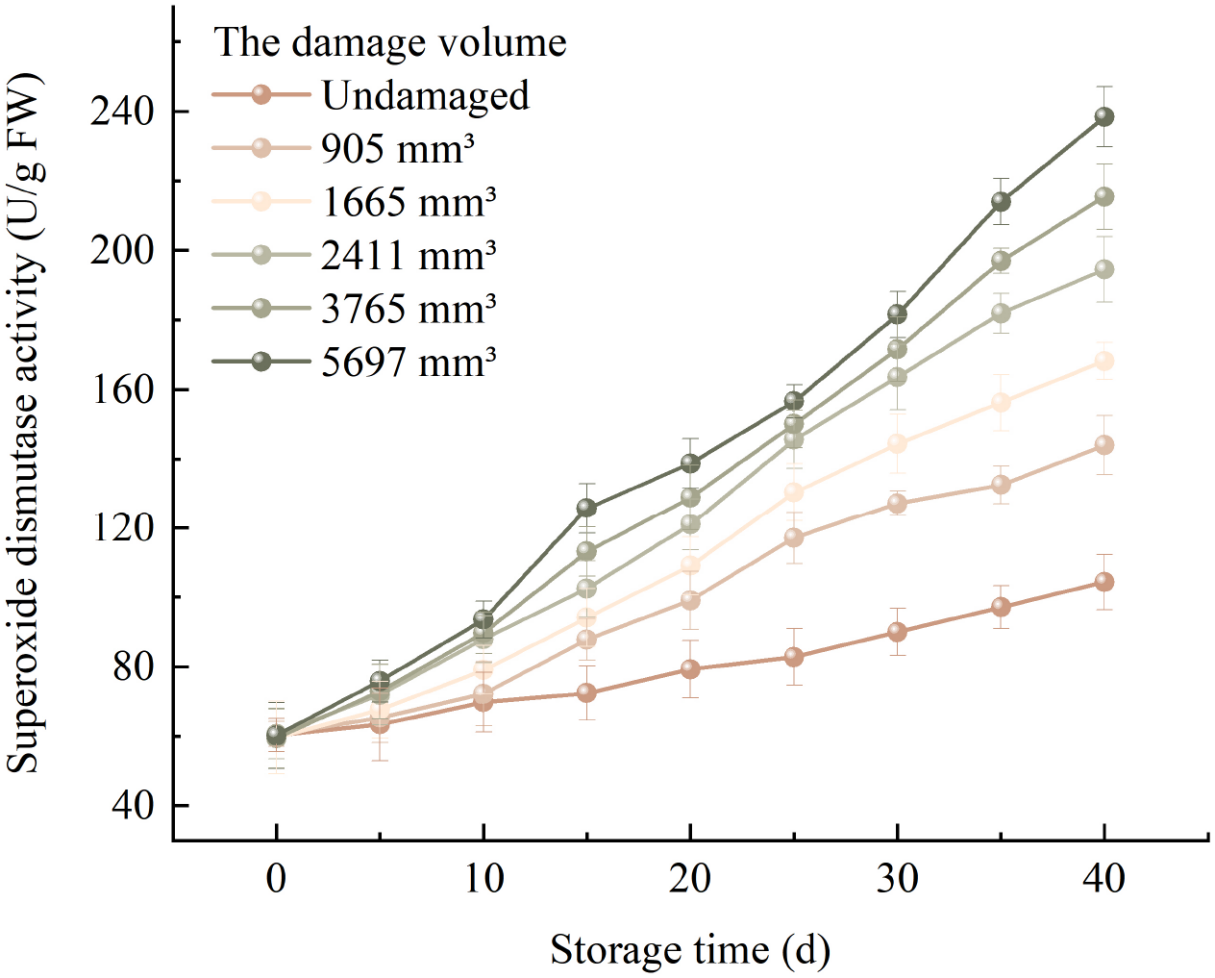
Trends in SOD activity of fragrant pears during storage.

The variation pattern of the average SOD activity of Korla fragrant pears with storage time under different damage degrees is shown in **Figure 3**. The average SOD activity of pears with different damage volumes gradually enhanced with the extension of storage time. The average SOD activity of undamaged pears enhanced from 60.274 U/g at 0 days of storage to 104.367 U/g after 40 days of storage. When the damage volume was 905mm^3^, the average SOD activity of damaged pears enhanced from 60.529 U/g to 143.957 U/g. The average SOD activity of pear samples with a damage volume of 1665mm^3^ enhanced from an initial 59.554 U/g to 168.229 U/g. The average SOD activity of pears with a damage volume of 2411mm^3^ enhanced from 60.579 U/g to 194.500 U/g. The average SOD activity of pears with a damage volume of 3765mm^3^ enhanced from 59.395 U/g to 215.434 U/g. The average SOD activity of pears with a damage volume of 5697mm^3^ enhanced from 60.211 U/g to 238.367 U/g. The results indicate that as the damage volume increased, the change rate of the average SOD activity of pears during the storage period gradually grew. The average SOD activity growth rate of pears with a damage volume of 5697mm^3^ was the fastest, and the growth speed of undamaged pears was the most slow.

### 3.2 Changes in catalase activity

**Figure 4:**
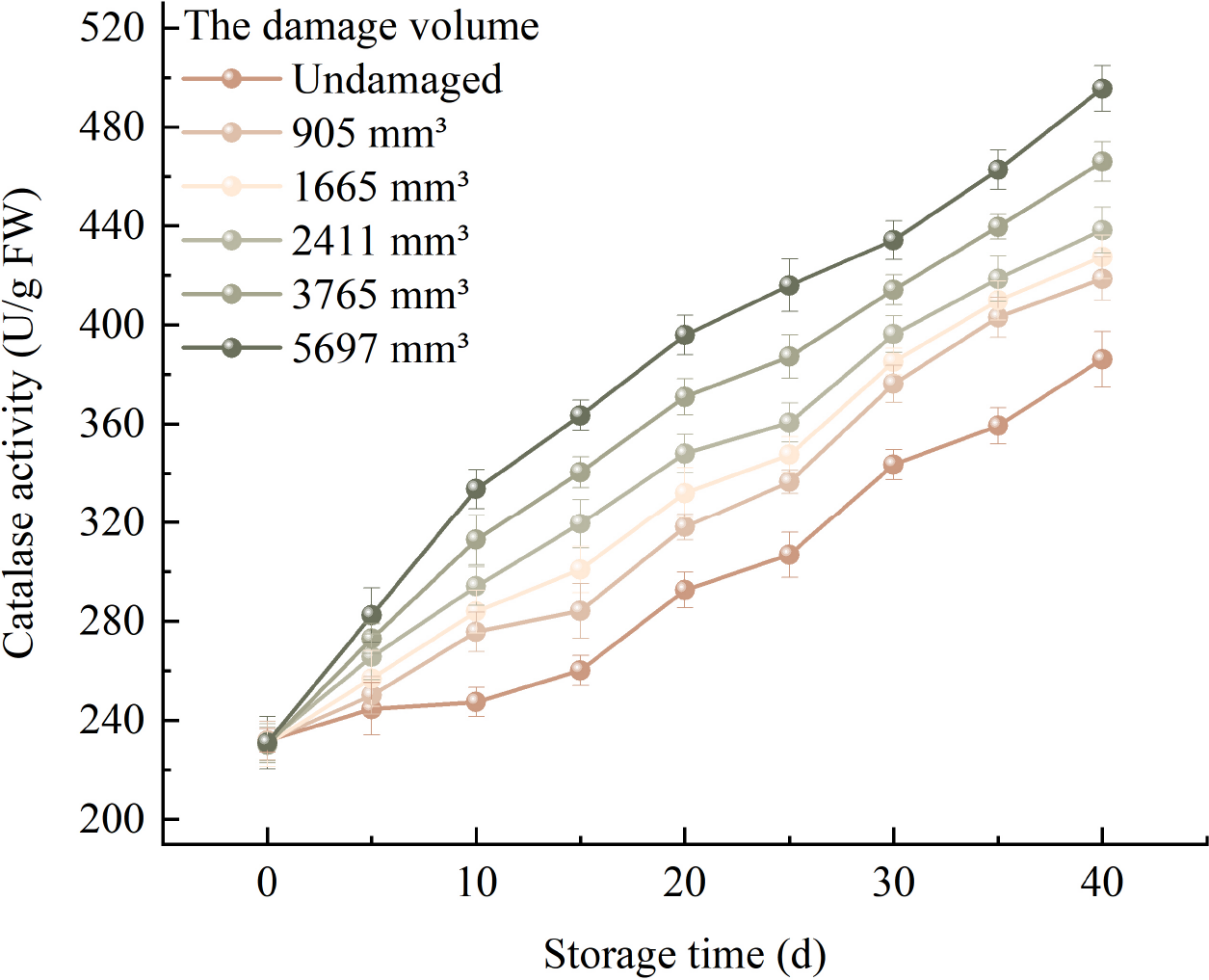
Trends in CAT activity of fragrant pears during storage.

**Figure 4** exhibits the variation patterns of the average CAT activity in fragrant pears with different damage levels during the storage period. The average CAT activity of fragrant pears with varying damage degrees gradually increased as the storage period extended. The average CAT activity of undamaged fragrant pears increased from an initial 231.799 U/g to 386.143 U/g after 40 days of storage. For pears with damage volumes of 905 mm^3^, 1665 mm^3^, 2411 mm^3^, 3765 mm^3^, and 5697 mm^3^, the average CAT activities at the beginning of storage were 231.783 U/g, 230.477 U/g, 231.111 U/g, 230.015 U/g, and 230.919 U/g, respectively. After 40 days of storage, these values reached 418.711 U/g, 427.535 U/g, 438.359 U/g, 465.899 U/g, and 495.439 U/g, respectively. The pear with a damage volume of 5697 mm^3^ exhibited the highest average CAT activity after 40 days of storage, while the undamaged pear showed the lowest. This indicates that as the damage volume increased, the rate of change in the average CAT activity of fragrant pears during storage progressively accelerated.

### 3.3 Changes in peroxidase activity

**Figure 5:**
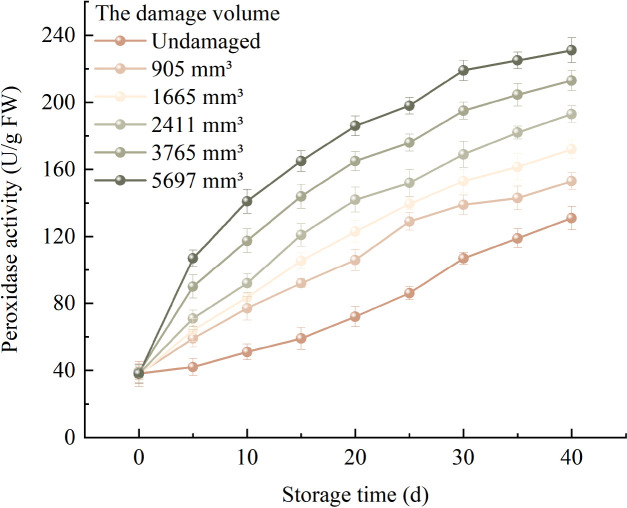
Trends in POD activity of fragrant pears during storage.

**Figure 5** shows the change pattern of the average POD activity of fragrant pears with different damage degrees during the storage period. The POD activity of fragrant pears with different damage degrees showed varying degrees of increase as the storage time extended. The average POD activity change rate of fragrant pears with a damage volume of 5697mm^3^ was the most obvious. The average POD activity after 40 days of storage enhanced from 38 U/g at the beginning of storage to 231 U/g. The average POD activity change of undamaged fragrant pears was the slowest. The average POD activity after 40 days of storage enhanced from the initial 38 U/g to 131 U/g. The average POD activity of fragrant pears with a damage volume of 905mm^3^ enhanced from 38 U/g to 153 U/g. The average POD activity of fragrant pears with a damage volume of 1665mm^3^ enhanced from 37 U/g to 172 U/g. The average POD activity of fragrant pears with a damage volume of 2411mm^3^ enhanced from 38 U/g to 193 U/g. The average POD activity of fragrant pears with a damage volume of 3765mm^3^ enhanced from 39 U/g to 213 U/g. The higher the damage degree, the greater the average POD activity change rate of fragrant pears during storage.

### 3.4 Changes in superoxide anion generation rate

**Figure 6:**
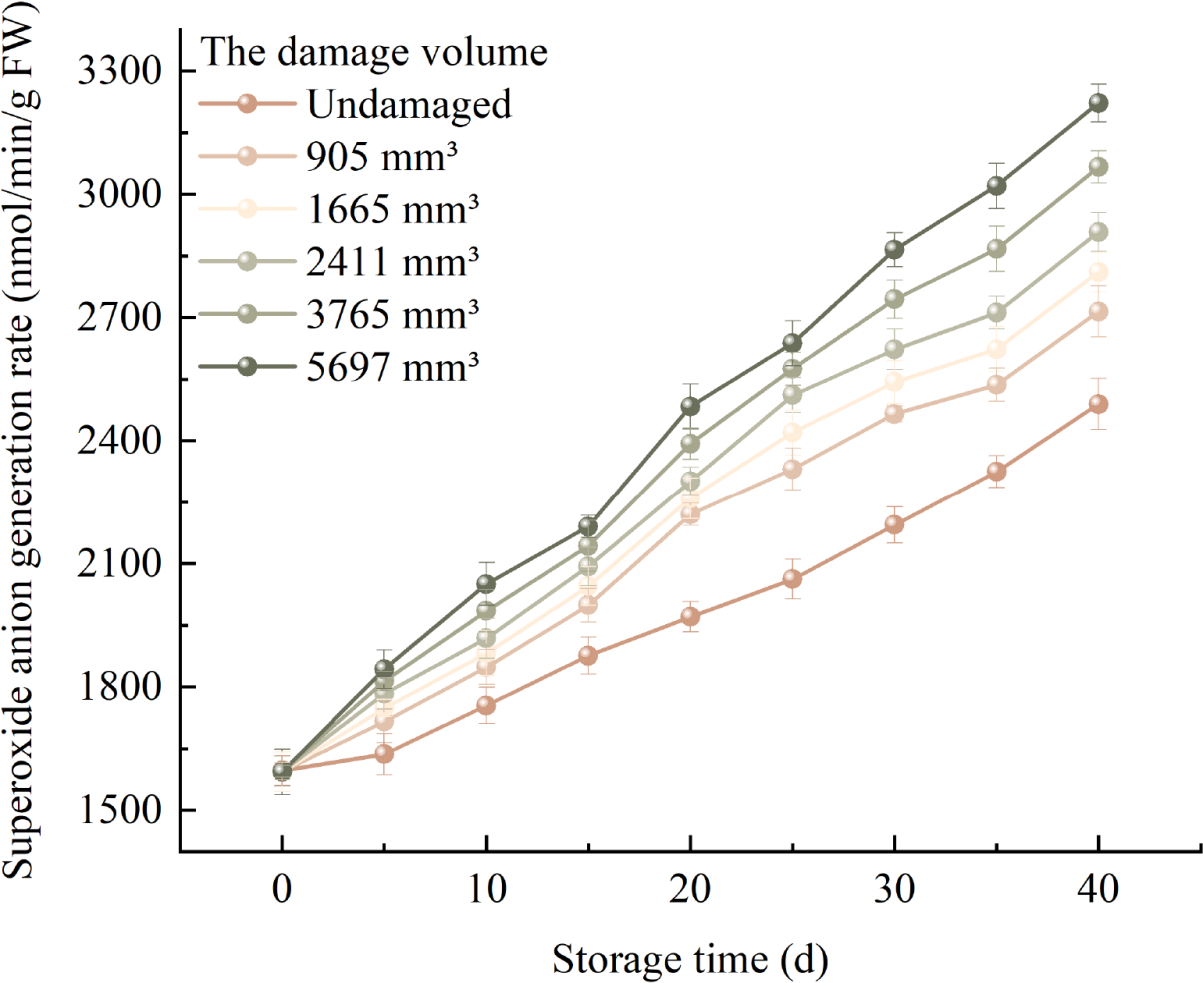
Trends in $O_{2}^{-.}$ generation rate of fragrant pears during storage.

The change pattern of the average $O_{2}^{-.}$ generation rate of fragrant pears under different damage degrees during the storage period is shown in **Figure 6**, the average $O_{2}^{-.}$ generation rate of fragrant pears under each damage volume gradually accelerated with the extension of storage time. At the same storage time, the average $O_{2}^{-.}$ generation rate of fragrant pears with 5697mm^3^ is the fastest, and the average $O_{2}^{-.}$ generation rate of undamaged fragrant pears is the slowest. The average $O_{2}^{-.}$ generation rate of undamaged fragrant pears after 40 days of storage increased from 1594.944 nmol/min/g at the beginning of storage to 2714.880 nmol/min/g, when the damage volume is 905mm^3^, the average $O_{2}^{-.}$ generation rate of fragrant pears increased from 1594.944 nmol/min/g at the beginning of storage to 2714.880 nmol/min/g, the average $O_{2}^{-.}$ generation rate of fragrant pears with a damage volume of 1665mm^3^ increased from 1593.832 nmol/min/g to 2810.648 nmol/min/g, the average $O_{2}^{-.}$ generation rate of fragrant pears with a damage volume of 2411mm^3^ increased from 1594.720 nmol/min/g to 2908.416 nmol/min/g, the average $O_{2}^{-.}$ generation rate of fragrant pears with a damage volume of 3765mm^3^ increased from 1595.024 nmol/min/g to 3066.072 nmol/min/g, the average $O_{2}^{-.}$ generation rate of fragrant pears with a damage volume of 5697mm^3^ increased from 1593.328 nmol/min/g to 3221.728 nmol/min/g. It indicates that the higher the damage degree, the fastest the growth rate of the average $O_{2}^{-.}$ generation rate of fragrant pears during the storage period.

### 3.5 Changes in hydrogen peroxide content

**Figure 7:**
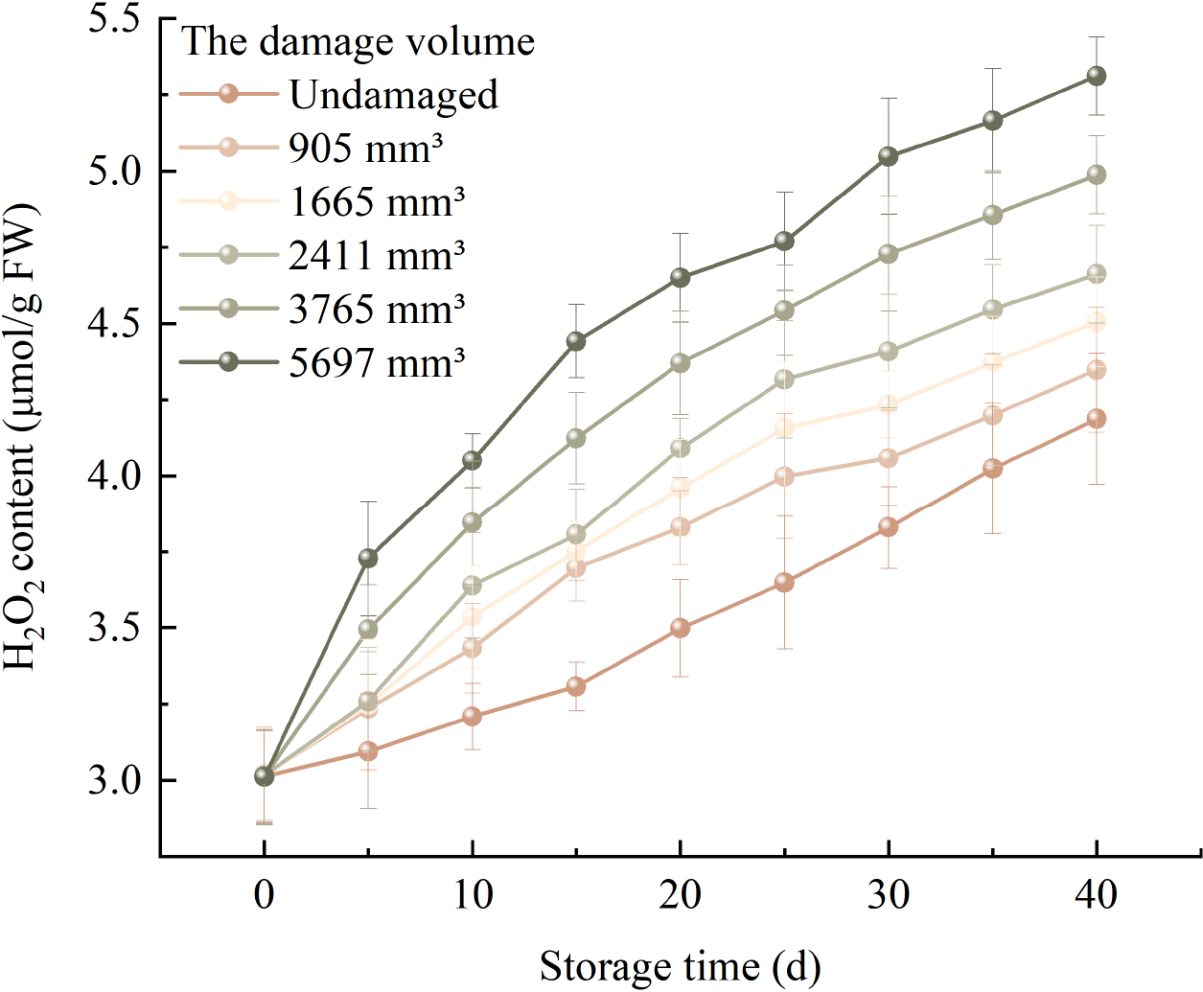
Trends in H_2_O_2_ content of fragrant pears during storage.

**Figure 7** illustrates the changes in the average H_2_O_2_ content of fragrant pears with different damage levels during storage. The average H_2_O_2_ content of pears across all damage volumes increased gradually with prolonged storage time, with higher damage levels correlating to faster rates of increase. Pears with a damage volume of 5697 mm^3^ exhibited the most rapid increase in average H_2_O_2_ content, rising from an initial 3.011 μmol/g to 5.312 μmol/g. For pears with damage volumes of 3765 mm^3^, 2411 mm^3^, 1665 mm^3^, and 905 mm^3^, the average H_2_O_2_ content increased from 3.014 μmol/g to 4.987 μmol/g, from 3.016 μmol/g to 4.662 μmol/g, from 3.018 μmol/g to 4.505 μmol/g, and from 3.019 μmol/g to 4.348 μmol/g, respectively. In contrast, undamaged pears showed the slowest rate of change, with the average H_2_O_2_ content increasing from an initial 3.014 μmol/g to 4.187 μmol/g.

This study investigated the changes in senescence quality of Korla fragrant pears with different damage levels under impact load during storage. The $O_{2}^{-.}$ generation rate and H_2_O_2_ content increased significantly with storage time, a trend consistent with the findings of Xu (Xu et al., 2022), Duan (Duan et al., 2011), and others in strawberries and lychees. This phenomenon occurs because ROS, such as $O_{2}^{-.}$, H_2_O_2_, gradually accumulate in plant cells during storage. Under impact damage stress, excessive accumulation of $O_{2}^{-.}$, H_2_O_2_ induces cell membrane peroxidation, thereby compromising membrane integrity (Bao et al., 2024). The browning observed at the damage sites is likely a result of disrupted cell membrane structures, which facilitates contact between POD and phenolic compounds (including flavonoids), ultimately leading to phenolic oxidation. The plant’s antioxidant system can neutralize ROS, mitigating the associated phytotoxic effects caused by their over-accumulation (Wang et al., 2019). SOD activity serves as the primary defense against oxidative stress by converting superoxide radicals into less toxic hydrogen peroxide. Consequently, SOD activity increased alongside the ethylene $O_{2}^{-.}$ generation rate (Scandalios, 1993). Both CAT and POD efficiently decompose hydrogen peroxide into water and oxygen, alleviating metabolic tissue damage induced by excessive H_2_O_2_ (Sekmen et al., 2007). Therefore, the activities of these antioxidant enzymes (SOD, CAT, and POD) increased throughout storage, promoting intracellular ROS balance, which aligns with observations by Ghosh et al. (2021) and Li et al. (2017) in peppers and bananas. Furthermore, under damage stress, phenolic compounds accumulated and contributed to cell wall reinforcement via lignification, thereby enhancing resistance to pathogen invasion and counteracting fruit senescence (Saini et al., 2024).

Mechanical damage leads to the release of compartmentalized enzymes and substrates, and wound stress promotes the production of phenolic compounds. These phenolic compounds are subsequently oxidized by polyphenol oxidase and POD into quinones, which then polymerize to form brown pigments, ultimately leading to fruit browning and senescence (Meng et al., 2025). Compared with undamaged Korla fragrant pears, those subjected to impact load damage exhibited faster $O_{2}^{-.}$ generation rate and a more rapid increase in H_2_O_2_ content within their cells. After 40 days of storage, the ROS content in the damaged pears was significantly higher. This indicates that mechanical damage promotes internal physiological responses in the fruit, accelerating the production of ROS and thereby inducing accelerated senescence and decay. In a previous study, Gu et al. (2022) reported similar findings, demonstrating that both the $O_{2}^{-.}$ generation rate and H_2_O_2_ content in sweet cherries with impact damage increased throughout the storage period and were significantly higher than those in undamaged cherries. This occurs because during storage, when the fruit experiences abiotic stress, internal electron flow reaches a high-energy state, combining with oxygen to form substantial amounts of ROS (Xu et al., 2022). Damage stress intensifies cellular oxidative stress, resulting in higher activities of SOD, CAT, and POD compared to undamaged pears. Furthermore, the rate of change in antioxidant enzyme activity increased progressively with greater damage volume. This is attributed to the rapid enhancement of antioxidant enzyme activity under abiotic stress, which helps counteract ROS-induced damage to components such as proteins. At higher damage levels, the accelerated accumulation of ROS within the pears necessitates a corresponding increase in antioxidant enzyme activity.

### 3.6 Prediction of senescence quality in pears during storage

To rapidly detect the senescence quality of damaged Korla fragrant pears during storage, this study developed a multi-output prediction model using PLSR, SVR, and LSTM to simultaneously predict SOD activity, CAT activity, POD activity, $O_{2}^{-.}$ generation rate, and H_2_O_2_ content. The model inputs were the damage volume and storage time of the pears, while the outputs consisted of the five senescence quality indicators. Seventy percent of all sample data were randomly selected as the training set for model development, with the remaining 30% reserved as the test set.

**Figure 8:**
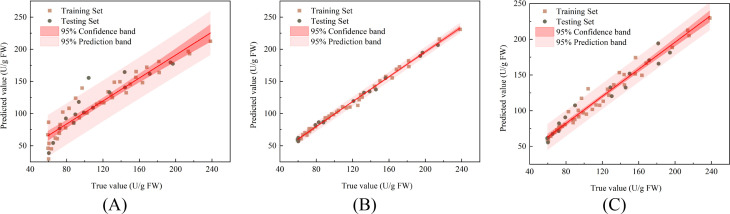
Correlation between predicted and measured SOD activity in fragrant pear **(A)** PLSR **(B)** SVR **(C)** LSTM.

The correlations between the actual values and predicted values of the PLSR, SVR, and LSTM models for predicting the SOD activity of damaged Korla fragrant pears during storage are shown in **Figures 8A–C**, respectively, along with the linear fitting trendlines and their 95% confidence bands and 95% prediction bands. The R^2^, RMSE, and RPD values for each model in both the training and testing sets are listed in **Table 1**. For predicting pear SOD activity, the PLSR model achieved R^2^, RMSE, and RPD values of 0.893, 15.909 U/g, and 3.098, respectively, in the training set, and 0.818, 18.135 U/g, and 2.419 in the testing set. The SVR model yielded R^2^, RMSE, and RPD values of 0.998, 2.139 U/g, and 23.986 in the training set, and 0.994, 3.924 U/g, and 13.623 in the testing set. The LSTM model produced R^2^, RMSE, and RPD values of 0.963, 8.844 U/g, and 5.277 in the training set, and 0.962, 9.519 U/g, and 5.299 in the testing set. Among these models, SVR demonstrated the best predictive performance for the SOD activity of damaged fragrant pears, exhibiting the highest R^2^ and RPD values, and the lowest RMSE.

**Table 1:** Prediction of pear senescence quality using PLSR, SVR, and LSTM multi-output models.

Quality indicator	Model	Training stage			Prediction stage		
		R^2^	RMSE	RPD	R^2^	RMSE	RPD
SOD activity (U/g FW)	PLSR	0.893	15.909	3.098	0.818	18.135	2.419
	**SVR**	**0.998**	**2.139**	**23.986**	**0.994**	**3.924**	**13.623**
	LSTM	0.963	8.844	5.277	0.962	9.519	5.299
CAT activity (U/g FW)	PLSR	0.966	13.911	5.454	0.970	11.362	5.995
	**SVR**	**0.997**	**3.999**	**17.310**	**0.995**	**5.656**	**14.684**
	LSTM	0.992	6.375	11.272	0.973	12.317	6.298
POD activity (U/g FW)	PLSR	0.931	15.003	3.851	0.903	16.336	3.317
	**SVR**	**0.997**	**3.027**	**18.524**	**0.994**	**3.718**	**13.516**
	LSTM	0.988	5.753	9.195	0.940	15.400	4.224
$O_{2}^{-.}$ generation rate (nmol/min/g FW)	PLSR	0.958	93.590	4.968	0.953	86.392	4.783
	**SVR**	**0.987**	**53.213**	**8.740**	**0.982**	**52.759**	**7.638**
	LSTM	0.989	46.420	9.436	0.976	72.027	6.693
H_2_O_2_ content (μmol/g FW)	PLSR	0.935	0.168	3.978	0.935	0.132	4.061
	**SVR**	**0.995**	**0.044**	**14.473**	**0.957**	**0.108**	**5.059**
	LSTM	0.991	0.055	10.829	0.954	0.145	4.821

The optimal prediction results of the model for each aging characteristic are highlighted in bold.

**Figure 9:**
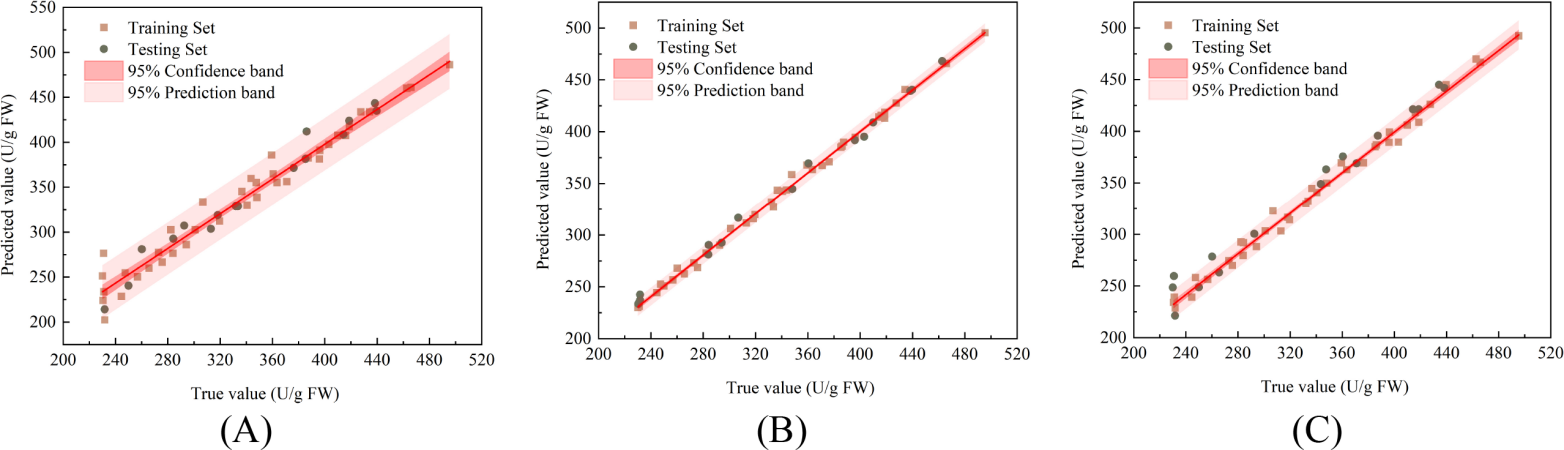
Correlation between predicted and measured CAT activity in fragrant pear **(A)** PLSR **(B)** SVR **(C)** LSTM.

**Figures 9A–C** show the correlation between the measured and predicted values of CAT activity in damaged Korla fragrant pears during storage, for the PLSR, SVR, and LSTM models during their training and prediction stages, respectively, along with the 95% confidence band and 95% prediction band. As presented in **Table 1**, for predicting CAT activity, the PLSR model achieved R^2^, RMSE, and RPD values of 0.966, 13.911 U/g, and 5.454, respectively, for the training set, and 0.970, 11.362 U/g, and 5.995 for the test set. The SVR model yielded R^2^, RMSE, and RPD values of 0.997, 3.999 U/g, and 17.310 for the training set, and 0.995, 5.656 U/g, and 14.684 for the test set. The LSTM model produced R^2^, RMSE, and RPD values of 0.992, 6.375 U/g, and 11.272 for the training set, and 0.973, 12.317 U/g, and 6.298 for the test set. The SVR model demonstrated the best performance in predicting the CAT activity of damaged pears. All models achieved R^2^ values exceeding 0.96 on the test set for this prediction task, indicating that the trained models can accurately predict CAT activity.

**Figure 10:**
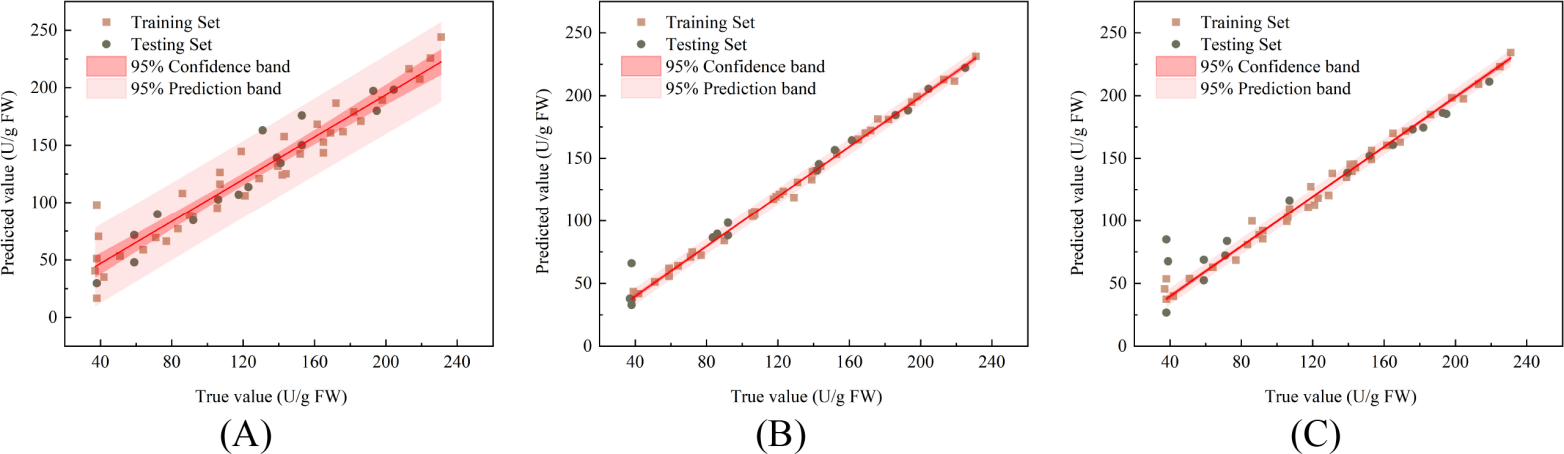
Correlation between predicted and measured POD activity in fragrant pear **(A)** PLSR **(B)** SVR **(C)** LSTM.

**Figures 10A–C** show the correlation between the measured and predicted values, along with the 95% confidence bands and 95% prediction bands, during both the training and prediction stages of the PLSR, SVR, and LSTM models for predicting POD activity in damaged fragrant pears during storage. The prediction results are summarized in **Table 1**. For predicting POD activity, the PLSR model achieved an R^2^, RMSE, and RPD of 0.931, 15.003 U/g, and 3.851, respectively, for the training set, and 0.903, 16.336 U/g, and 3.317 for the test set. The SVR model yielded an R^2^, RMSE, and RPD of 0.997, 3.027 U/g, and 18.524 for the training set, and 0.994, 3.718 U/g, and 13.516 for the test set. The LSTM model resulted in an R^2^, RMSE, and RPD of 0.988, 5.753 U/g, and 9.195 for the training set, and 0.940, 15.400 U/g, and 4.224 for the test set. Among these models, SVR demonstrated the best prediction performance for the POD activity of damaged Korla fragrant pears.

**Figure 11:**
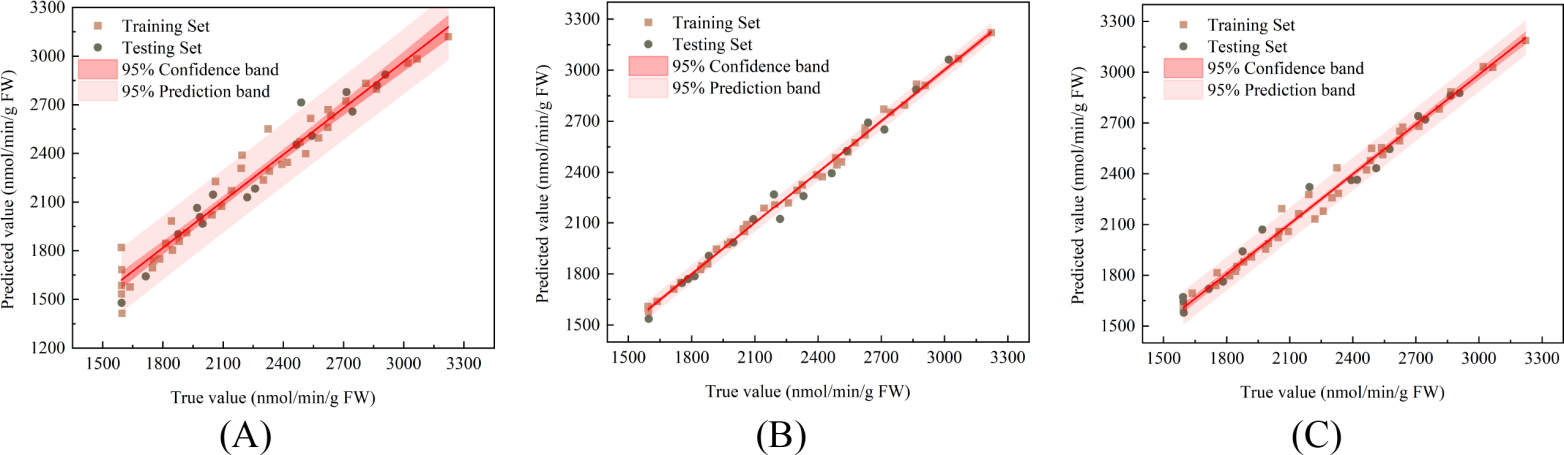
Correlation between predicted and measured $O_{2}^{-.}$ generation rate in fragrant pear **(A)** PLSR **(B)** SVR **(C)** LSTM.

The correlations between the actual and predicted values for the PLSR, SVR, and LSTM models in predicting the $O_{2}^{-.}$ generation rate of damaged Korla fragrant pears during storage are presented in **Figures 11A–C** for the training and prediction stages, respectively. The linear regression trend lines are shown, along with their corresponding 95% confidence bands and 95% prediction bands. The prediction results are presented in **Table 1**. For the PLSR model in predicting the $O_{2}^{-.}$ generation rate, the R^2^, RMSE, and RPD for the training set were 0.958, 93.59 nmol/min/g, and 4.968, respectively, while for the test set, they were 0.953, 86.392 nmol/min/g, and 4.783. For the SVR model, the R^2^, RMSE, and RPD in the training set were 0.987, 53.213 nmol/min/g, and 8.740, respectively, and in the test set, they were 0.982, 52.759 nmol/min/g, and 7.638. For the LSTM model, the R^2^, RMSE, and RPD in the training set were 0.989, 46.420 nmol/min/g, and 9.436, respectively, and in the test set, they were 0.976, 72.027 nmol/min/g, and 6.693. The SVR model demonstrated the best prediction performance for the $O_{2}^{-.}$ generation rate of damaged pears, followed by the LSTM and PLSR models.

**Figure 12:**
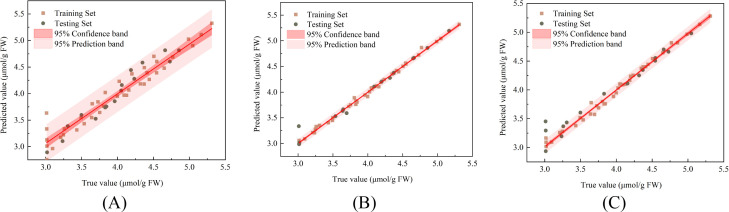
Correlation between predicted and measured H_2_O_2_ content in fragrant pear **(A)** PLSR **(B)** SVR **(C)** LSTM.

**Figures 12A–C** respectively illustrate the correlations between the measured and predicted values of H_2_O_2_ content in damaged Korla fragrant pears during storage, along with the 95% confidence bands and 95% prediction bands, during the training and prediction phases of the PLSR, SVR, and LSTM models. The prediction results of the models on both the training and test sets are shown in **Table 1**. For the PLSR model predicting H_2_O_2_ content, the training set yielded R^2^, RMSE, and RPD values of 0.935, 0.168 μmol/g, and 3.978, respectively, while the test set results were 0.935, 0.132 μmol/g, and 4.061. The SVR model achieved R^2^, RMSE, and RPD of 0.995, 0.044 μmol/g, and 14.473 on the training set, and 0.957, 0.108 μmol/g, and 5.059 on the test set. The LSTM model produced respective R^2^, RMSE, and RPD values of 0.991, 0.055 μmol/g, and 10.829 for the training set, and 0.954, 0.145 μmol/g, and 4.821 for the test set. The SVR model demonstrated superior performance in predicting the H_2_O_2_ content of damaged pears, indicating that the trained SVR model can accurately forecast H_2_O_2_ content.

## 4 Discussion

Korla fragrant pears are highly susceptible to mechanical damage during harvesting, storage, and transportation. Impact damage is the most severe type of mechanical damage (Liu et al., 2024). However, some damaged pears still retain storage value and can be utilized as raw materials for further processing, such as in juice or canned products. This study developed simultaneous prediction models for SOD activity, CAT activity, POD activity, $O_{2}^{-.}$ generation rate, and H_2_O_2_ content in fragrant pears during storage. The constructed SVR-based simultaneous prediction model achieved an average R^2^ of 0.984 in predicting various senescence-related quality parameters. Compared with single-component prediction models, the simultaneous detection method can acquire multiple fruit quality indicators at once, significantly improving prediction efficiency and reducing model development and maintenance costs. By capturing and leveraging the hidden synergistic relationships among these indicators, the overall prediction performance was enhanced. In previous research, Zhao et al. (2025) implemented a multi-output structure based on a Gaussian process regression model for the synchronous detection of tannin and protein content in sorghum. Their model achieved an R^2^ of 0.979, RMSE of 0.0587, and RPD of 6.8928 for predicting tannin content, and an R^2^ of 0.950, RMSE of 0.1699, and RPD of 4.4710 for predicting protein content. Zhang et al. (2024) developed a one-dimensional convolutional neural network based on channel and spatial attention mechanisms to establish a synchronous detection method for the four main catechin contents in tea. The R^2^ values for predicting epicatechin, epicatechin gallate, epigallocatechin, and epigallocatechin gallate in tea were all greater than 0.90. Gabbitas et al. (2023) implemented synchronous detection of three common mycotoxins in maize using a label-free surface-enhanced Raman spectroscopy approach combined with multivariate statistical analysis. The results demonstrated correlation coefficients of 0.74, 0.89, and 0.72 for predicting aflatoxin B1, zearalenone, and ochratoxin A, respectively. In comparison, the SVR proposed in this study for assessing senescence quality in damaged fragrant pears demonstrates superior predictive performance. This may be attributed to the fact that the SVR multi-output model is more adept at handling nonlinear relationships and small sample data, effectively capturing the intrinsic correlations and dependencies among different output variables. By introducing the “ε-insensitive loss function” and the “kernel trick,” it exhibits excellent robustness and flexibility in addressing regression problems (Tran et al., 2024).

However, the current research has certain limitations. For example, this study only provides a preliminary investigation into the changes in the senescence quality of fragrant pears subjected to impact damage. During the harvesting, transportation, and subsequent processing of the fruit, damage to Korla fragrant pears can occur not only from impact loads but also from compressive loads, vibration, and even the interactions between these various loads. The damage characteristics of Korla fragrant pears under other types of loads and the interactions between them require further in-depth investigation. In addition, this study preliminarily analyzed the correlation between ROS and phenolic metabolism, and explored the potential role of phenolic metabolism in fruit browning and senescence. Furthermore, the study of quality deterioration in fragrant pears during storage was conducted at room temperature. Storage in cold rooms or under other conditions may slow the process of fruit quality deterioration. However, specific influence patterns still require further investigation. Subsequent research will further investigate the quality evolution of Korla fragrant pears during storage under different damage types or storage conditions. The impact and underlying mechanisms of other metabolic pathways, such as phenolic compounds, on fruit senescence will also be explored. Extending this method to the quality detection of other perishable fruits or other key indicators (such as phenolic compounds), and promoting the intelligent and precise development of postharvest agricultural product management, holds significant practical implications for enhancing the modernization of the fruit and vegetable industry, reducing postharvest waste, and ensuring market supply.

## 5 Conclusions

In Korla fragrant pears with different damage levels, the SOD activity, CAT activity, POD activity, $O_{2}^{-.}$ generation rate, and H_2_O_2_ content all increased to varying degrees during storage. Pears with higher damage levels exhibited faster changes in senescence quality. The constructed SVR multi-output model demonstrated the best performance in predicting the SOD activity, CAT activity, POD activity, $O_{2}^{-.}$ generation rate, and H_2_O_2_ content in damaged pears. On the test set, the R^2^ values were 0.994, 0.995, 0.994, 0.982, and 0.957, respectively. The RMSE values were 3.924 U/g, 5.656 U/g, 3.718 U/g, 52.759 nmol/min/g, and 0.108 μmol/g, respectively. And the RPD values were 13.623, 14.684, 13.516, 7.638, and 5.059, respectively. The SVR multi-output model developed in this study can accurately and efficiently predict the senescence quality of fragrant pears during storage. This model significantly enhanced the efficiency and comprehensiveness of fruit quality detection by enabling the simultaneous prediction of five senescence quality indicators. It aids relevant practitioners in precisely determining the senescence state of damaged pears, thereby providing technical support for optimizing postharvest storage techniques and quality control. In subsequent research, the predictive effectiveness of this method for the quality of other fruit types will be further validated.

## Data Availability

The original contributions presented in the study are included in the article/supplementary material. Further inquiries can be directed to the corresponding author.
